# Qualitative research in oral health in India: A bibliometric analysis

**DOI:** 10.1016/j.jobcr.2025.11.001

**Published:** 2025-11-10

**Authors:** Abhishek Mehta, Barsha Priya Deka, Manu Raj Mathur

**Affiliations:** a Department of Public Health Dentistry, Faculty of Dentistry, Jamia Millia Islamia, New Delhi, 110025, India; b Health Policy, Public Health Foundation of India (PHFI), Gurugram, Haryana, 122 002, India

**Keywords:** Qualitative research, Mixed-method research, Bibliometric analysis, Dentistry, India, Oral health

## Abstract

**Background:**

A qualitative research approach is necessary to understand the complexities of social, behavioral, and systemic factors influencing oral health. Despite having a large dental education infrastructure and workforce, a limited number of studies have been published by Indian dental researchers utilizing a qualitative methodology.

**Objective:**

Map the existing literature to understand the current landscape of qualitative research in oral health in India.

**Method:**

A comprehensive search of prominent databases, along with handsearching, was conducted to shortlist studies based on a predefined criterion. Eligible studies underwent thematic categorization and bibliometric assessment. Keyword co-occurrence and network analysis were performed using VOSviewer software to identify conceptual linkages and emerging research themes.

**Results:**

Sixty-four studies met the inclusion criteria. Of these, 56 % were pure qualitative and 43 % were mixed-methods studies, with most published in the last five years. Key focus areas include tobacco cessation, pediatric oral health, and barriers to accessing dental services. Methodologically, the studies relied heavily on interviews and focus group discussions, with limited theoretical integration. Thematic clusters showed dominance of behavior-focused research and regional disparities in study distribution.

**Conclusion:**

This is the first review to comprehensively chart the evolution of qualitative research in oral health in India. While momentum is growing, significant gaps remain in methodological depth and thematic diversity. Strengthening training, institutional support, and theoretical engagement is essential for advancing qualitative dental research in India.

## 1 Introduction

A range of social, political, and economic factors affects the oral health of a person or population group. These macro environmental conditions encompass where an individual is born, lives, socializes, and works, collectively referred to as the Social Determinants of Oral Health. These determinants influence people's behavior towards oral health and access to oral care services.^1^ Global trends in oral diseases present a paradoxical situation, while overall prevalence is declining in most of the population groups within High-Income Countries (HICs), it remains disproportionately high among the most disadvantaged and marginalized communities. In contrast, Low- and Middle-Income countries (LMICs) are witnessing a surge in the incidence of all major oral diseases. This skewed distribution creates inequalities in oral health among different socioeconomic groups within and between countries, highlighting a global public health challenge.^2^

Research focused solely on exploring the quantitative impact of traditional risk factors of oral diseases such as sugar intake, tobacco consumption, and oral hygiene practices, provides limited insight into the underlying cause of these inequalities.^3^ To effectively understand and address these oral health inequalities, we need to examine the broader determinants, including sociocultural norms, gender- and age-related vulnerabilities, political and commercial influences, and the accessibility and affordability of oral health services. Traditionally, oral health research in LMICs has primarily focused on quantitative methods such as epidemiological surveys using clinical indices or structured questionnaires to assess oral health behavior and practices. While these approaches are useful for quantifying disease burden, their limitations include a lack of ability to capture the felt and expressed needs of a population and to explore complex interpersonal, psychosocial, and socio-environmental factors that shape oral health outcomes.^4^

Qualitative research has its origins in early 20th-century sociology and anthropology studies. It was originally designed to understand human behavior in natural contexts through methods like ethnography and participant observation. Over time, it was adopted by health sciences to explore the complex social, cultural and experiential dimensions of health and illness. ^5^^,^^6^It seeks to uncover how people interpret their health experiences and navigate healthcare systems, using tools such as interviews, focus groups, observations and thematic or narrative analysis. Qualitative research follows a systematic path of knowledge generation. It begins with framing a research question, grounded in clear objectives and often informed by theory-building strategies. It involves identification of main themes, followed by coding the data to classify elements into subcategories or themes. These classifications evolve through characterization, where connections and relationships between concepts are mapped out to assess their significance. Finally, explanation integrates findings into plausible interpretations that may contribute to hypotheses or broader theoretical propositions. This progression from raw data to theoretical insights illustrates how qualitative investigation builds towards theory rigorously and transparently.^7^ In Dentistry, it's important to understand that dental care is not only influenced by biomedical factors but also by psychosocial behaviors, doctor-patient communication, health-seeking behaviors, affordability and societal norms. Barriers like fear, past poor experience, misinformation from community networks, out-of-pocket expenditure or regional disparities in oral health literacy can deter individuals from seeking necessary care.^7^ To uncover these underlying causes and lived realities behind the rising oral health burden, especially in LMICs like India, there is a pressing need to conduct more qualitative studies or incorporate qualitative methodologies alongside traditional data-driven approaches for a more holistic understanding through a mixed-methods approach. Given this context and the increasing relevance of qualitative inquiry in addressing oral health disparities, it is imperative to examine how such research has evolved in oral health in India. Therefore, we conducted a comprehensive review of literature and bibliometric analysis to systematically investigate publication trends, thematic orientations, methodological frameworks, and collaborative patterns in qualitative and mixed-method dental research conducted in India.

## 2 Methodology

A review of existing scientific literature and bibliometric analysis was conducted to systematically identify the publication trends, areas, gaps in current knowledge and inform future directions in qualitative dental research in India. Pre-registration of review protocol was done in Open Science Framework. (reg. no.- 10.17605/OSF.IO/PSU5Q).

### 2.1 Search strategy

A comprehensive literature search was conducted up to May 2025 on PubMed, Scopus, and Web of Science using a combination of keywords and MeSH terms. The search strategy for PubMed was: “qualitative study" [Title/Abstract] OR "qualitative research" [Title/Abstract] OR "mixed methods" [Title/Abstract] OR "interview" [Title/Abstract] OR ″ Focus group discussion" [Title/Abstract] OR "qualitative research" [MeSH Terms] OR "qualitative analysis" [Title/Abstract]) AND "dentistry" [MeSH Terms] OR "oral health" [MeSH Terms] OR "tooth" [MeSH Terms] OR "tobacco cessation" [Title/Abstract] OR "dental [Title/Abstract] OR "oral health research" [Title/Abstract] AND “India" [MeSH Terms] OR "Indian population" [Title/Abstract]. This search string was then adapted for use in the other two databases. The search was limited to peer–reviewed original research articles conducted in India, with no restrictions on the year of publication.

### 2.2 Inclusion and exclusion criteria

Original research articles published in one of the above three databases and utilizing a qualitative or mixed-methods approach to study an oral health-related topic among an Indian population were eligible for inclusion in this review. The main reasons for exclusion included cross-sectional studies that employed a structured or semi-structured questionnaire without a qualitative component, studies unrelated to oral health, studies conducted on overseas Indian populations, and review articles. A PRISMA flowchart was prepared to illustrate the selection process.^8^

### 2.3 Data extraction and analysis

Two reviewers (AM, BPD) independently screened the titles, abstracts, and full-texts of all retrieved articles. Any discrepancies or uncertainties during the screening or data extraction process were discussed, and when consensus could not be reached, a third reviewer (MM) was consulted to provide a final decision. All final study inclusion decisions were made based on agreement among all three reviewers. The full text of all the eligible studies was screened and relevant data were extracted and systematically organized into a standardized excel spreadsheet under the headings like study title, authors, year of publication, journal, type of research (qualitative or mixed methods), data collection methods, data analysis techniques, main themes identified and number of citations (from PubMed, Scopus and Web of science). Descriptive statistics were performed to present data by year of publication, type of research (qualitative or mixed-methods), data collection methods, data analysis techniques, main themes identified and number of citations in PubMed, Scopus and Web of Science.

### 2.4 Bibliometric analysis

We conducted a bibliometric analysis of published peer-reviewed literature to assess publication trends in research utilizing qualitative or mixed-methods approaches. This was achieved by measuring publication volume over time, evaluating research impact through citation counts, and performing network analysis to visualize collaboration patterns while identifying emerging research topics using frequently used keywords and thematic clusters. To explore thematic linkage and conceptual structure across the included studies, a term co-occurrence analysis was conducted using VOSviewer software 1.6.20.^9^

Steps followed for this Analysis were:

1. *Choosing Type of Data:* We selected "Create a map based on text data" to build a term co-occurrence network from the titles and abstracts of all included studies.
2. *Choosing Data Source:* The option "Read data from reference manager file" was chosen. The source file was exported in. RIS/endnote format, which is compatible with EndNote, RefWorks, and other reference managers.
3. *Selecting Fields for Term Extraction:* Terms were extracted from the Title and Abstract fields. The options to ignore structured abstract labels (e.g., Background, Methods, Results) and ignore copyright statements were both selected to ensure clean term extraction.
4. *Choosing the Counting Method:* We selected Full Counting, where each term's co-occurrence is given equal weight regardless of how frequently it appears across documents.
5. *Keyword Selection Criteria:* A minimum threshold of 1 occurrence per term was applied.
6. *Network Map Interpretation:* The final co-occurrence network was generated based on the strength of associations between keywords, where Nodes (circles) represent individual keywords, and the Size of the node reflects the frequency of occurrence; the larger the node, the more frequently the keyword appeared in the data set. Lines (edges) between nodes indicate co-occurrence relationships. The thickness and color intensity of a line denote the strength of co-occurrence; stronger connections imply more frequent co-appearance in titles/abstracts, and colors represent distinct clusters**,** each highlighting a major thematic area based on keyword groupings.

## 3 Results

**Fig. 1:**
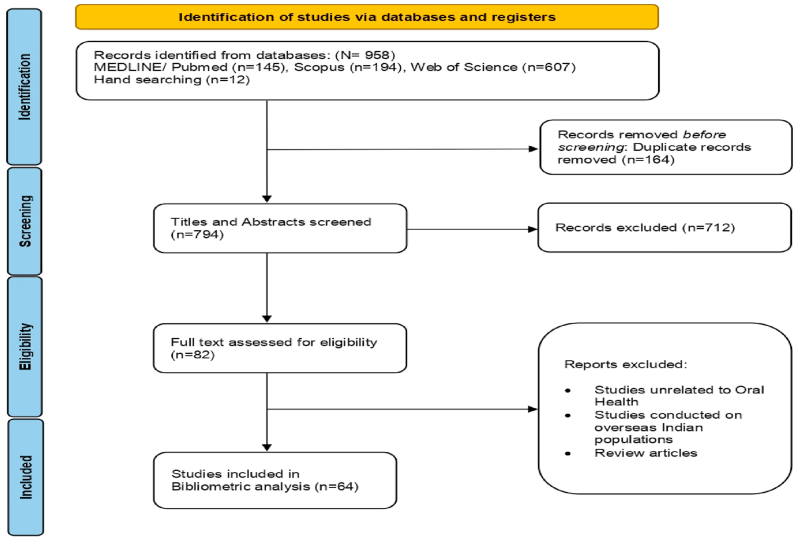
PRISMA flowchart outlining the search strategy.

A total of 958 citations were found from three prominent databases (PubMed = 145, Scopus = 194, Web of Science = 607) and Hand searching (n = 12). The search results were exported into Rayaan software for further screening and removal of duplicates (n = 164). The screened titles and abstracts of the remaining 792 citations to shortlist eligible studies for full text reading and final analysis. A total of 64 studies were found eligible.^10^, ^11^, ^12^, ^13^, ^14^, ^15^, ^16^, ^17^, ^18^, ^19^, ^20^, ^21^, ^22^, ^23^, ^24^, ^25^, ^26^, ^27^, ^28^, ^29^, ^30^, ^31^, ^32^, ^33^, ^34^, ^35^, ^36^, ^37^, ^38^, ^39^, ^40^, ^41^, ^42^, ^43^, ^44^, ^45^, ^46^, ^47^, ^48^, ^49^, ^50^, ^51^, ^52^, ^53^, ^54^, ^55^, ^56^, ^57^, ^58^, ^59^, ^60^, ^61^, ^62^, ^63^, ^64^, ^65^, ^66^, ^67^, ^68^, ^69^, ^70^, ^71^, ^72^, ^73^, ^74^ The visual representation outlines the screening and selection process is presented as a PRISMA flowchart (Fig. 1).

### 3.1 Descriptive profile of the included studies

Each included study was categorized by publication characteristics, research designs, population groups, and thematic areas. It was observed that over half of the studies were purely qualitative (n = 36, 56 %).^10^, ^11^, ^12^^,^^15^^,^^16^^,^^18^^,^^20^, ^21^, ^22^, ^23^, ^24^, ^25^, ^26^, ^27^, ^28^, ^29^, ^30^, ^31^, ^32^, ^33^, ^34^^,^^43^^,^^44^^,^^46^, ^47^, ^48^, ^49^^,^^51^^,^^52^^,^^54^^,^^56^, ^57^, ^58^, ^59^, ^60^, ^61^, ^62^, ^63^, ^64^, ^65^^,^^67^^,^^68^^,^^71^^,^^73^ They primarily focused on understanding experiences, perceptions, and social phenomena through in-depth interviews and thematic analysis. The remaining 28 studies (43 %) 13, 14, 15^,^^17^^,^^19^^,^^35^, ^36^, ^37^, ^38^, ^39^, ^40^, ^41^, ^42^^,^^45^^,^^50^^,^^53^^,^^55^^,^^69^^,^^72^^,^^74^ followed a mixed-methods design, referred to as a research design that combines both qualitative and quantitative data collection and analysis techniques. Interview methods (semi-structured, in-depth) and focus group discussions were the most frequently used data collection techniques, while thematic analysis was the dominant analytical approach.^6^(Table 1).

**Table 1:** Summary of the included studies in the Bibliometric analysis.

S. No.	Authors	Title	Year of Publication	Journal Name	Type of Research (Qualitative Study or Mix method)	Population Studied	Data Collection Method	Data Analysis Method	TheoreticalFramework, if any	Main Themes Identified	Number of Citations (Scopus/PubMed)
1	Sharath Asokan, GeethaPriya P. Ramakrishnan, Kirthi Varshini Ranganatham, Sudhandra Viswanath, Shyam Sivasamy^36^	Stress amongst paediatric dental postgraduate students in India: A mixed-method approach	2022	European Journal of Dental Education	Mixed Method	Paediatric dental postgraduate students in India	Surveys and focus group discussions	Quantitative statistical analysis and thematic analysis	Not specified	Academic pressure, clinical workload, coping mechanisms	0
2	Aswathy Sreedevi, Anindo Majumdar, Yvonne Olando, Marie Chan Sun, Catriona Jennings, Kemi Tibazarwa, Holly Grey, Katarzyna Zatonska, Rinu Pk, Shana Shirin Najeeb^54^	Experiences and Beliefs on Tobacco Use, Cessation in India: A Qualitative Study	2023	Global Health	Qualitative	Individuals in India who use tobacco	In-depth interviews	Thematic analysis	Not specified	Cultural perceptions of tobacco use, barriers to cessation, awareness of health risks	0
3	Kamal Shigli, S Jyotsna, G Rajesh, Umesh Wadgave, Banashree Sankeshwari, Sushma S Nayak, Rashmi Vyas^18^	Challenges in Learning Preclinical Prosthodontics: A Survey of Perceptions of Dental Undergraduates and Teaching Faculty at an Indian Dental School	2017	Journal of Clinical and Diagnostic Research (JCDR)	Qualitative	Undergraduate dental students and prosthodontics faculty at an Indian dental school	Focus Group Discussions	Thematic analysis	A phenomenological approach to explore lived experiences and perceptions	The vastness and complexity of prosthodontics making it difficult to visualize and correlate theory with practice. Lack of clinical exposure and reliance on conventional teaching methods hindering understanding.	12
4	Himanshu A. Gupte, M. D'Costa, S. Ramanadhan, K. Viswanath^10^	Factors Influencing Implementation of a Workplace Tobacco Cessation Intervention in India: A Qualitative Exploration	2021	Workplace Health & Safety	Qualitative	Facilitators (program coordinators and counselors) of a workplace tobacco cessation intervention in India	In-depth interviews	Thematic analysis guided by the Consolidated Framework for Implementation Research (CFIR)	Consolidated Framework for Implementation Research (CFIR)	Promoters and barriers to implementing a workplace tobacco cessation program, including factors like management engagement, adaptability of intervention components, peer support among employees, and advocacy for tobacco-free policies	6
5	Shah R, Shah R, Shah S, Bhojani U^11^	Integrating tobacco cessation into routine dental practice: Protocol for a qualitative study	2019	BMJ Open	Qualitative (Study Protocol)	Oral health professionals (OHPs) and dental patients in Ahmedabad, Gujarat, India	Individual interviews with OHPs and dental patients	Individual interviews with OHPs and dental patients	Not specified	The study aims to understand the functioning of the oral healthcare system towards tobacco cessation and capture the views of dental patients on tobacco cessation services provided by OHPs	6
6	Supriya Lahoti, Rajmohan Panda, Rajath R. Prabhu, Sangeeta Das, Sithun Kumar Patro, Irwin Nazareth^55^	Validation of Mobile Messages for an mHealth Intervention for Smokeless Tobacco Cessation in India	2023	Asian Pacific Journal of Cancer Prevention	Mixed-method	13 patients who were tobacco users (smokeless or dual users) at urban primary health centers in Berhampur, Odisha, India	Quantitative ratings using a Likert scale for clarity and appeal of 32 mobile messages; in-depth interviews guided by a 5-item discussion guide	Framework analysis	Trans-theoretical Model (TTM)	User-centered message development, Preference for audio concise messages, high clarity and appeal scores, effective message domains, finalization of message domains	1
7	Lakshmi Puzhankara, Vineetha Karuveettil, Chandrashekar Janakiram, Ramprasad Vasthare, Sowmya Srinivasan, Angel Fenol^13^	Exploring medical and dental practitioner perspectives and developing a knowledge attitude and practice (KAP) evaluation tool for the common risk factor approach in managing non-communicable and periodontal diseases	2024	BMC Oral Health	Mixed Method	Medical and dental practitioners in South India	Qualitative: Online interviews with medical and dental practitioners, Quantitative: Structured KAP (Knowledge, Attitude, Practice) questionnaire	*Qualitative*: Thematic analysis, Quantitative: Statistical analysis using standardized scoring; internal consistency measured with Cronbach's alpha	Common Risk Factor Approach (CRFA)	Understanding of common risk factors, Risk factor reduction and disease burden, Integrating CRFA into clinical practice, Barriers to CRFA implementation.	0
8	Priyanka Athavale, Nehaa Khadka, Shampa Roy, Piyasree Mukherjee, Deepika Chandra Mohan, Bathsheba (Bethy) Turton, Karen Sokal-Gutierrez^14^	Early Childhood Junk Food Consumption, Severe Dental Caries, and Undernutrition: A Mixed-Methods Study from Mumbai, India	2020	International Journal of Environmental Research and Public Health	Mixed Method	Children aged 6 months to 6 years and their mothers in low-income urban communities in Mumbai, India	Quantitative data were collected through interviews with mothers (n = 959) regarding maternal-child nutrition and oral health, along with dental examinations and anthropometric assessments of the children.	Descriptive and logistic regression analyses were performed on quantitative data, while content analysis was conducted on qualitative data	Not specified	Nutrition transition and junk food environment, Prevalence and severity of early childhood caries, Association between ECC and undernutrition, barriers to oral health care and policy and programmatic implications.	20
9	Priyanka Ravi, Deepali Aggarwal, Bharathi M. Purohit, Upendra Singh Bhadauria, Harsh Priya^56^	Qualitative Analysis of Opinions and Beliefs Associated with the Use of Tobacco Dentifrice among Individuals Attending a Tobacco Counseling Session	2023	Asian Pacific Journal of Cancer Prevention	Qualitative	Individuals using tobacco dentifrice attending a tobacco counseling session at a dental hospital in India	In-depth interviews with 30 participants	Thematic analysis	Not specified	The study identified six categories: reasons for initiation (family, peers, curiosity), awareness of health effects, perceptions of tobacco dentifrice use, perceptions on quitting, reasons for continued use, and societal use. Participants often believed in myths regarding the benefits of tobacco dentifrice for dental and systemic health	0
10	Ashwini Manish Dadpe, Dipali Yogesh Shah, Vineet Vinay, Pratibha Shetkar^20^	Factors Facilitating Academic Success in Dental Students After Initial Failure: A Qualitative Study	2018	Journal of Dental Education	Qualitative	Dental students at Sinhgad Dental College and Hospital, Maharashtra, India, who had previously failed written examinations but subsequently achieved academic success	Semi-structured interviews based on Appreciative Inquiry (AI) with 15 participants selected through purposive sampling	Thematic analysis	Appreciative Inquiry (AI)	The study identified four key themes that facilitated academic success among dental students after initial failure: 1.Learning Strategies: Students modified their attitudes toward studying, adopting a more learning-oriented approach.2.Resources.3.Psychological Aspects.4.Environmental Factors	8
11	Priyanka Ravi, Anupama Ivaturi, Diptajit Das, Upendra Singh Bhadauria, Charu Khurana, Monica Dev, Harsh Priya^37^	Evaluation of Perceptions of Tobacco Cessation among the Individuals Attending a Tertiary Care Dental Hospital – A Mixed Methods Design	2021	Asian Pacific Journal of Cancer Prevention	Mixed Method	Individuals attending a tertiary care dental hospital in India	*Quantitative:* Self-administered questionnaires assessing current and past tobacco use, dependence (using Fagerström Test for Nicotine Dependence for both smoking and smokeless tobacco), and quit attempt,Qualitative: Face-to-face interviews exploring perceptions on tobacco quitting	Quantitative: Descriptive statistics.Qualitative: Thematic analysis	Transtheoretical Model (Stages of Change)	Most participants had tried quitting and were in the contemplation stage, motivated by health concerns and social support. Some lacked awareness of cessation methods and believed smokeless tobacco was less harmful.	1
12	Diana Constance, Rohini Subbiah, Aparna Sukumaran, Parangimalai Diwakar Madankumar^21^	Barriers in Maintaining Oral Health Among Children with Cerebral Palsy – Parent/Caregiver's Perspective	2023	Journal of Indian Society of Pedodontics and Preventive Dentistry	Qualitative	Parents and caregivers of children with cerebral palsy in Chennai, Tamil Nadu, India	In-depth interviews	Thematic analysis	Not specified	Oral health care for children with cerebral palsy was hindered by physical limitations, caregiver knowledge gaps, financial constraints, limited access to specialized clinics, and caregiver stress.	0
13	Pollachi-Ramakrishnan, G.P., Asokan, S., Balaraman, C., Viswanath, S., Dhamodharan, Y.K.T.^22^	Pediatrician's Perception of Oral Health in Children – A Qualitative Study	2023	Journal of Indian Society of Pedodontics and Preventive Dentistry	Qualitative	Pediatricians practicing in Western Tamil Nadu, India	Face-to-face semi-structured interviews	Grounded theory approach with thematic analysis of transcribed interviews	Grounded Theory	Pediatricians recognized oral health's importance but cited limited training, time constraints, and lack of referral guidelines as barriers, while showing willingness to engage if supported with resources and training.	1
14	B.S. Suprabha, R. Shenoy, K.Y. Mahabala, A.P. Nayak, A. Rao, V. D'Souza^23^	Early Childhood Caries and Dental Care Utilization in Mangalore, India: Parents' Perceptions	2024	JDR Clinical & Translational Research	Qualitative	Parents of children with early childhood caries (ECC) seeking dental care in Mangalore, India	Focus group discussions	Manual line-by-line coding and content analysis	Not specified	Dental Care Visiting Patterns,Significance of Dental Visits,Challenges to Dental Care Utilization,	0
15	Abhijit Nadkarni, Leena Gaikwad, Miriam Sequeira, Richard Velleman, Joseline D'souza, Ankita Hoble, RajanishHaldankar^52^	Evaluation of Feasibility and Acceptability of a Text-Messaging Intervention for Tobacco Cessation in India	2023	Nicotine & Tobacco Research	Qualitative	Individuals using smoked and smokeless tobacco in India	Semi-structured interviews and focus group discussions	Thematic analysis	Not specified	Feasibility,Acceptability,Barriers,Suggestions were made to tailor messages to individual needs and to include voice messages for those with literacy challenges	0
16	Ar Dongre, Pradeep Deshmukh, N. Murali, B.S. Garg^53^	Tobacco consumption among adolescents in rural Wardha: where and how tobacco control should focus its attention	2008	Indian Journal of Cancer	Mixed Method	Adolescents aged 15–19 years in rural Wardha, India	Community-based survey with house-to-house visits and focus group discussions	tatistical analysis for quantitative data; thematic analysis for qualitative data	Not specified	Prevalence of Tobacco Use,Types of Tobacco Products Used,Reasons for Tobacco Use, Tobacco Use Among Girls	48
17	GeethaPriya PR, Sharath Asokan, Sudhandra Viswanath^35^	Job Satisfaction and Stress Among Dental Faculty Members: A Mixed-Method Approach	2021	Journal of Dental Education	Mixed Method	Dental faculty members in Tamil Nadu, India	mixed-method approach.	Quantitative data were analyzed using Mann–Whitney, Kruskal–Wallis, and Friedman tests. Qualitative data were analyzed through thematic analysis	Not specified	Insights into the perceptions and experiences of dental faculty members regarding their profession. Potential Stressors, Sequelae of Stress,Stress-Free Job	8
18	Darshan Chauhan, Yogesh Murugan, Dhruv Patel, Nidhi Trivedi^42^	Health literacy and tobacco cessation among hypertensive individuals: A mixed-method study	2024	Journal of Education and Health Promotion	Mixed Method	305 hypertensive adults attending an urban tertiary hospital in Gujarat, India	Interviewer-administered questionnaires for quantitative data; in-depth interviews for qualitative data	Descriptive statistics and multivariable logistic regression for quantitative data; thematic analysis for qualitative data	Not specified	Prevalence of Tobacco Use, Socio-Demographic Factors, Triggers for Tobacco Use, Barriers to Cessation, Facilitators for Cessation	0
19	Rajmohan Panda, Supriya Lahoti, Arti Mishra, Rajath R. Prabhu, Sangeeta Das, Durga Madhab Satapathy, Irwin Nazareth^12^	Designing a Mobile Health Smokeless Tobacco Cessation Intervention in Odisha, India: User and Provider Perspectives	2023	Digital Health	Qualitative	Smokeless tobacco (SLT) users and healthcare providers in Berhampur, Odisha, India	In-depth interviews (IDIs) with 26 SLT users and 5 primary care physicians; focus group discussions (FGDs) with 2 counselors	Framework analysis method	Transtheoretical Model (TTM) of behavior change	Current Scenario of SLT Use, Barriers and Facilitators for Quitting SLT, Design and Delivery of the Proposed Intervention,	0
20	B.S. Suprabha, R. Shenoy, K.Y. Mahabala, A.P. Nayak, A. Rao, V. D'Souz^23^	Early Feeding and Weaning Practices of Indian Children with Early Childhood Caries: A Qualitative Exploration	2023	JDR Clinical & Translational Research	Qualitative	Parents of children with early childhood caries (ECC) seeking dental treatment at an academic dental college in India	Focus group discussions with 27 parents using a structured discussion guide	Content analysis	Not specified	Influence of Community Norms,Risky Feeding Practices,Challenges in Weaning,Knowledge-Action Gap	2
21	S. Naganandini, T. Seemadevi, Alexander Maniangat Luke, Mohamed Saleh Hamad Ingafou^24^	Smoking Trends and Awareness Among Indian University Students: A Qualitative Study	2024	Heliyon	Qualitative	Indian university students	In-depth interviews and focus group discussions	Thematic analysis	Not specified	Smoking Initiation, Motivators and Barriers to Quitting, Perception of Tobacco Cessation Centers (TCCs)	0
22	Jyothi Tadakamadla, Santhosh Kumar, Ratilal Lalloo, Newell W. Johnson^25^	Qualitative analysis of the impact of Oral Potentially Malignant Disorders on daily life activities	2017	PLOS ONE	Qualitative	Patients diagnosed with Oral Leukoplakia, Oral Submucous Fibrosis, and Oral Lichen Planus attending the Oral Medicine clinic of Panineeya Institute of Dental Sciences & Research Centre, Hyderabad, India	Eighteen in-depth interviews and three focus group discussions conducted in a non-clinical setting; voice recordings were transcribed and translated from Telugu to English	Thematic analysis using NVivo software	Not specified	Difficulties with Diagnosis and Knowledge About the Condition,Physical Impairment and Functional Limitations,Psychological and Social Wellbeing,Effects of Treatment on Daily Life	48
23	Arti Singh and Shikha Dixit^26^	Exploring barriers of quitting smokeless tobacco among coronary artery disease patients of India: A qualitative study	2021	Chronic Illness	Qualitative	Twelve patients diagnosed with coronary artery disease (CAD) who were current users of smokeless tobacco (SLT) in India	Semi-structured interviews conducted with the participants	Thematic analysis	Not specified	Socio-environmental Support for SLT Use,Misconceptions About SLT and Cardiac Health, Low Self-Efficacy to Quit, Fatalistic Attitudes, Substitution with Perceived Less Harmful Products	3
24	Garima Bhatt, Sonu Goel, Sandeep Grover, Bikash Medhi, Nidhi Jaswal, Sandeep Singh Gill, Gurmandeep Singh^27^	Feasibility of tobacco cessation intervention at non-communicable diseases clinics: A qualitative study from a North Indian State	2023	PLOS ONE	Qualitative	Healthcare providers (Medical Officers, Counselors, Program Officers, Nurses) at NCD clinics in Punjab, India	in-depth interviews	Framework method of qualitative analysis, focusing on six areas: acceptability, demand, adaptation, practicality, implementation, and integration	Not specified	Appropriateness and Suitability, Actual Use of Intervention Activities, Extent of Delivery to Intended Participants, Barriers and Favorable Factors, Modifications Required, Integrational Perspectives	2
25	K. Koka, S. Yadlapalli, P. Pillarisetti, M. Yasangi, A. Yaragani, S. Kummamuru^28^	The barriers for tobacco cessation counseling in teaching health care institutions: A qualitative data analysis using MAXQDA software	2021	Journal of Family Medicine and Primary Care	Qualitative	133 house surgeons (dental interns) from two teaching dental institutions in Andhra Pradesh, India	Focus group discussions	Thematic analysis	Not specified	Student tobacco used, pateient reluctant, lack of qualification	1
26	Anne Stoebner-Delbarre and Mira B. Aghi^29^	A comparative study of perceptions on tobacco in vulnerable populations between India and France	2013	Global Health Promotion	Qualitative	60 disadvantaged women residing in South Delhi, 163 adults with disabilities residing in the south of France	Focus group discussions	Thematic analysis	Not specified	Daily Life Circumstances as Reasons for Tobacco Use, Barriers to Quitting, Lack of Awareness About Nicotine Dependence, Health Risk Awareness, Demand for Professional Support	5
27	Anju James, Shwetha Kanyaloor Mallikarjuna, Pushpanjali Krishnappa^38^	Perception of Caregivers about Oral Health Services for Institutionalized Elderly – A Mixed Method Study	2024	Annals of Geriatric Medicine and Research	Mixed Method	54 institutionalized older adults and 54 caregivers from old age homes in Bengaluru, India	Quantitative- questionnaire, Qualitative - Indepth	Quantitative: Descriptive statistical,Qualitative: Thematic analysis	Not specified	Lack of Cooperation from Older Adults, Financial Constraints, Lack of Knowledge, Time Constraints,	0
28	Soumita Ghose, Aseem Mahajan, Soumitra Shankar Datta^30^	A public policy analysis with key stakeholders' insights to understand India's compliance with the WHO framework convention on tobacco control	2022	ecancermedicalscience	Qualitative	Nine experts from diverse professional backgrounds, including preventive oncology researchers, tobacco cessation specialists, public health experts, clinicians, and human rights activists across various regions of India	In-depth qualitative interviews with nine experts, Systematic review of 14 research papers,	Thematic analysis, Qualitative, Health policy analysis comparing India's national tobacco control policies with the WHO Framework Convention on Tobacco Control (WHO-FCT	Walt and Gilson's Policy Triangle Framework, which examines policy content, context, actors, and processes	Conflicting Government Policies, Influence of the Tobacco Industry, Demand Reduction Strategies, Legislation on Second-hand Smoke, Health Warnings and Packaging, Advertising and Promotion, Youth Protection Measures	1
29	Anisha Rodrigues, Shradha Dhanania, and Rohan Rodrigues^31^	If I have teeth, I can smile.” Experiences with tooth loss and the use of a removable dental prosthesis among people who are partially and completely edentulous in Karnataka, India	2021	BDJ Open	Qualitative	Individuals who are partially and completely edentulous in Karnataka, India	in-depth interviews	Thematic analysis	Not specified	Perception of Tooth Loss, Psychosocial Impact, Functional Challenges, Prosthesis Use, Financial Constraints	3
30	Sneha Malhotra, Vikrant Mohanty, Aswini Y. Balappanavar, Vaibhav Gupta, Shivam Kapoor, Surbhi Kapoor^39^	Stakeholder perspectives on the integration of oral health into national health schemes: A mixed-method study research design in Delhi, India	2021	Journal of Family Medicine and Primary Care	Mixed Methods	96 stakeholders, including (MOs), (ASHAs), (ANMs), and dental surgeons from nine Delhi Government dispensaries	Questionnaires and Focus group discussions	Descriptive statistical analysis and Thematic analysis	Not specified	Positive Attitude Towards Integration, Barriers to Integration, Barriers to Integration,Need for Training and Support	1
31	SaanuSidharthan, VenkitachalamRamanarayanan, Vineetha Karuveettil, Greeshma C. Ravindran^19^	Utilization of dental health services and its associated factors among adult population in Ernakulam district, Kerala, India: A mixed-method analysis	2024	Journal of Oral Biology and Craniofacial Research	Mixed Methods	544 adults aged 18 years and above from urban and rural wards of Ernakulam district, Kerala, India	Questionnaires and Focus group discussions, indepth interviews	Quantitative: Univariate and multivariate statistical analyses, Qualitative: Thematic analysis	Andersen's Behavioral Model of Health Services Use	Low Utilization Rates,AssociatedFactors,FinancialConstraints,Negative Attitudes	4
32	Chandrashekar B R, Nishath Khanum, Praveen Kulkarni, Madhu Basavegowda, Kishor M, Suma S^41^	E-learning module on tobacco counseling for students of medicine and dentistry in India: a needs analysis using mixed-methods research	2024	BMJ Public Health	Mixed Methods	Final-year MBBS and BDS students, as well as subject experts from medical and dental colleges in Southern India	In-depth interviews and questionnaires	Thematic analysis, Descriptive statistics	Grounded Theory approach within a modified exploratory sequential mixed-methods design	Curricular Gaps, Departmental Silos, Limited Competence, Need for E-learning Modules	0
33	D. Anusha, S. Keingadarane, D. J. Caplan, S. Sivasamy^33^	Exploring the obstacles affecting the oral health of adolescents with intellectual disabilities: insights from maternal perspectives—a qualitative study	2025	European Archives of Paediatric Dentistry	Qualitative	22 mothers of adolescents aged 12–18 years with intellectual disabilities in Pondicherry, India	Semi-structured interviews	Thematic analysis	Not specified	Physical and Behavioral Challenges, Limited Access to Specialized Dental Care, Financial Constraints, Lack of Professional Support and Education, Emotional and Psychological Impact on Mothers, Social Stigma and Isolation	1
34	Himanshu A. Gupte, Marina D'Costa, Shilpi Gupta, Vinayak Sonawane^34^	Integration of a Tobacco Cessation Program into a Rural Community-Based Maternal and Child Health Program in India: A Stakeholders' Perspective on Task Shifting	2024	Nicotine & Tobacco Research	Qualitative	Stakeholders involved in (MCH) programs in rural India, including (CHWs), program managers, and healthcare providers	In-depth interviews and focus group discussions	Thematic analysis	Not specified	Feasibility of Task Shifting, Training and Capacity Building, Supportive Supervision, Community Engagement	1
35	Rajmohan Panda, Divya Persai, Manu Mathur, Bidyut Kanti Sarkar^15^	Perception and Practices of Physicians in Addressing the Smokeless Tobacco Epidemic: Findings from Two States in India	2013	Asian Pacific Journal of Cancer Prevention	Mixed Methods	Physicians practicing in two high smokeless tobacco (SLT) prevalence states in India	in-depth interviews, Questionnaires	Thematic analysis, Descriptive statistics	Not specified	Limited Knowledge of SLT Health Effects, Barriers to SLT Cessation Counseling, Need for Capacity Building	1
36	Soumita Ghose, Alok Sardar, Suman Shiva, Brega Ellen Mullan, Soumitra S. Datta^16^	Perception of tobacco use in young adults in urban India: a qualitative exploration with relevant health policy analysis	2019	ecancer	Qualitative	Young adults (university students) in urban India	Interviews and focus group discussions	Thematic analysis	Health policy analysis framework	Social and Behavioral Factors, Lack of Awareness, Perceived Ineffectiveness of Tobacco Control Measures, Need for Comprehensive Tobacco Cessation Support	11
37	Nadia Claire Mascarenhas E Dias, Vishakha Uday Kamble, Jagadish Anil Cacodca^17^	Clinico-epidemiological profile and barriers to cessation among tobacco users attending a tobacco cessation clinic in Goa	2025	Journal of Family Medicine and Primary Care	Mixed Methods	224 tobacco users attending a tobacco cessation clinic in Goa	Questionnaires and Focus Group Discussion	Descriptive and Thematic analysis	Not specified	Early Initiation, Peer Influence, Lack of Prior Attempts to Quit, Barriers to Cessation	1
38	Jena S, Kumar G, Tripathi R, Khandelwal S, Sharma O, Arora S^44^	The Barrier to Accessing Dental Healthcare Services Among the Institutionalized Visually Impaired Adults: A Qualitative Study	2024	Curues	Qualitative	Institutionalized Visually impaired adults in India	In-Depth interviews	Thematic analysis	Not specified	Lack of awareness, financial constraints, fear and anxiety, lack of caregiver support, accessibility issues	0
39	Kumari M, Sharma S, Raj A, Jha A, Shivakumar S, Kumar A^45^	Addressing Oral Health Disparities of a Tribal Population Through a Combined Implementation of Focus Group Discussion, Mobile Technology Networking, and Creating a Supportive Environment: A Prospective Study	2023	Curues	Mixed Methods	Tribal Population In India	Focus Group Discussion	Descriptive and Thematic analysis	Not specified	Oral health awareness, digital outreach, community engagement, environmental support	0
40	Goswami S, Gupta SS, Raut A^46^	Understanding the Psychosocial Impact of Oral Cancer on the Family Caregivers and their Coping up Mechanism: A Qualitative Study in Rural Wardha, Central India	2019	Indian Journal of Palliative Care	Qualitative	Family caregivers of oral cancer patients in rural Wardha, India	In-Depth interviews	Thematic analysis	Not specified	Emotional distress, economic burden, spiritual coping, family support, role strain	12
41	Purohit BM, Singh A, Barbi W, Ahmad S^48^	Cultural factors and family influences on adolescent oral health: qualitative research in a socially disadvantaged population	2024	International Journal of Paediatric Dentistry	Qualitative	Adolescents from socially disadvantaged communities and their family	In-Depth interviews and focus group discussions	Thematic analysis	Not specified	Cultural beliefs, family influence, health-seeking behavior, economic constraints, oral health awareness	0
42	Srinivas R, Anandakrishna L^49^	Parental issues and concerns for their children treated under general anaesthesia for early childhood caries: A qualitative research approach	2021	Indian Journal of Dental Research	Qualitative	Parents of children treated under general anaesthesia for early childhood carie	In-Depth interviews	Thematic analysis	Not specified	Emotional anxiety, financial burden, trust in healthcare system, postoperative care concerns	3
43	Anagha KA, Megha M, Karuveettil V, Vijay Kumar S^40^	Perceptions of barriers towards dental appointment keeping among patients of a tertiary care setting: A mixed method exploration	2024	Journal of Oral Biology and Craniofacial Research	Mixed Methods	Patients attending a tertiary care dental facility in India	In-Depth interviews and structured questionnaire	Descriptive and Thematic analysis	Not specified	Forgetfulness, work commitments, fear/anxiety, logistical challenges, lack of awareness	0
44	Singh S, Savana K, Brajendu, Priya P, Jain AK, Kumar A^50^	Psychological Effects of Orthodontic Treatment in Adults: A Mixed-Methods Study	2024	Journal of Pharmacy &Bioallied Sciences	Mixed Methods	Adult orthodontic patients in India	Questionnaires and semi-strucutred interviews	Descriptive and Thematic analysis	Not specified	Improved self-esteem, treatment-related anxiety, social perceptions, expectations from treatment	0
45	Shenoy R, Das D, Mukherjee M, Baranya Shrikrishna S, Denny C, D'Souza V^57^	Dentists' perceptions on present and future dental practice during the COVID-19 pandemic: An embedded study	2022	F1000Research	Qualitative	Practicing dentists in India during COVID-19	Online semi-structured interviews	Thematic analysis	Not specified	Practice uncertainty, adaptation to protocols, financial concerns, mental health impact, tele-dentistry outlook	5
46	Palaniraja S, Taghavi K, Kataria I, Oswal K, Vani NV, Liji AA, Parekh H, Isaac R, Kuriakose M, Swaminathan R, Rebello R, Purushotham A, Basu P, Sullivan R, Chandran A^47^	Barriers and contributions of rural community health workers in enabling cancer early detection and subsequent care in India: a qualitative study	2025	BMC Public Health	Qualitative	Rural community health workers involved in cancer care in India	In-depth interviews and focus group discussions	Thematic analysis	Theoretical Framework method	Service barriers, CHW contributions, training gaps, community trust, patient follow-up, referral challenges	0
47	Rodrigues A, Hegde V, Hegde A V, Shastri SG, Ravikumar DN, Rodrigues R.^32^	An exploration of the oral health beliefs and behaviors of people living with HIV in Mangalore, India: a qualitative study.	2021	BMC Oral Health	Qualitative	Rural community health workers involved in cancer care in India	In-depth interviews and focus group discussions	Thematic analysis	Theoritical Framework method	Service barriers, CHW contributions, training gaps, community trust, patient follow-up, referral challenges	0
48	Kumar S, Adhikari P, Thoppil S, George B, Jose L, Meenakshi K^58^	Dietary practices among children with early childhood caries and the associated factors: A qualitative study	2021	International Journal of Paediatric Dentistry	Qualitative	Parents of children with early childhood caries	In-depth interview	Thematic analysis	Not specified	Parental knowledge and beliefs, Feeding practices and routines, Sugar consumption and its role in dental health, Barriers to healthy dental care, Cultural and social influences	27
49	Suprabha BS, D'Souza V, Shenoy R, Karuna YM, Nayak AP, Rao A^59^	Early childhood caries and parents' challenges in implementing oral hygiene practices: a qualitative study	2021	International Journal of Paediatric Dentistry	Qualitative	Parents of children with early childhood caries (six focus groups)	Focus group discussion, Audio recordings, Field notes	Content analysis	Not specified	Knowledge gap on selecting proper oral hygiene aids, Barriers in implementing routines at home, Awareness without proper application	13
50	Shigli K, Nayak S, Lagali-Jirge V, Nerali JT, Vadavi D, Oginni FO, Kusurkar RA^60^	Dental teachers' perceptions about the status of geriatric dentistry in the dental curriculum: A qualitative exploration in the Indian context	2025	Gerodontology	Qualitative	Dental teachers (Heads of Department, Board of Study members, Deans, Lecturers, Associate Professors) in India	Four focus group discussions, audio-recorded, transcribed verbatim	Thematic analysis with inductive coding, consensus through deliberation	Not specified	Unique challenges in managing geriatric patients, dental care, attitude, awareness, social issues, trategies for advancement (sensitisation of policymakers, collaboration, curriculum modification, improved educational strategies)	0
51	Shetty P, Srivastav R (corrected to Srivastava R), Debnath A, Murali R, Shamala A^61^	The tobacco trade and trail in Karnataka	2018	Indian Journal of Cancer	Qualitative	Tobacco farmers in Mysore district; tobacco vendors in Bangalore	Semi-structured interviews with audio recording	Inductive content analysis	Not specified	Awareness of tobacco laws vs poor compliance; attitudes toward tobacco ban	1
52	Murthy V, Sethuraman KR, Choudhury S, Shakila P^62^	Application of Practice Oriented-Peer Review for Prosthodontics (PRO-PReP) – A Qualitative Study	2021	International Journal of Psychiatry in Medicine	Qualitative descriptive	Prosthodontic postgraduates	Periodic peer-review meetings, group discussion,video observation, feedback sessions	Thematic analysis combined with video-recorded reactions using Kalamazoo scale	Integrated R2C2 model (Relationship-Reaction-Content-Coaching) and Balint method	Improved confidence in patient communication, enhanced self-awareness, effective peer learning, positive change in professional behavior	2
53	Esther K, Muthu MS, Sagarkar AR, Saikia A^63^	Content Analysis of Brief Telephonic Conversation with Parents of Children with Cleft Lip and Palate During Sustained Anticipatory Guidance Sessions	2024	Cleft Palate Craniofacial Journal	Qualitative	Parents/caregivers of children with cleft lip and palate (8 children, 40 calls)	40 brief telephone calls; recorded, transcribed verbatim	Thematic content analysis	Not specified	General health concerns, Oral health & development, Surgery-related questions,Emotional support; plus observed positive shift in parental oral health behaviours	0
54	Aurlene N, Aravinth V, Balan I N, Kote S^64^	Expounding on the concerns of Indian politicians regarding fluorosis: A qualitative analysis of parliamentary questions on fluorosis over two decades	2021	Indian Journal of Public Health	Qualitative	Parliamentary questions (Lok Sabha & Rajya Sabha) from 1999 to 2019	Document analysis of 107 parliamentary questions	Inductive thematic analysis using NVivo 12 & Braun & Clarke framework	Not specified	Health hazards of fluoride exposure; Fluorosis control measures; Magnitude/prevalence of fluorosis in India	3
55	Priyanka Athavale, Kristin Hoeft, Rupal M. Dalal, Ameya P. Bondre, Piyasree Mukherjee, Karen Sokal-Gutierrez^65^	A qualitative assessment of barriers and facilitators to implementing recommended infant nutrition practices in Mumbai, India	2020	Journal of Health, Population and Nutrition	Qualitative	Caregivers (mothers and paternal grandmothers) of children aged 0–2 years in two slum communities, Mumbai	semi-structured, in-depth interviews, audio-recorded and transcribed verbatim	Qualitative content analysis using software	Not specified	Barriers: lack of nutrition knowledge, conflicting messages, limited social support, low maternal self-efficacy; Facilitators: professional nutrition guidance, family support, maternal empowerment	16
56	Swati Jain, Vikrant Mohanty, Shekhar Grover^66^	Tobacco legislation perception and barriers: A qualitative insight towards tobacco free schools in Delhi, India	2021	Tobacco Induced Diseases	Qualitative (phenomenology)	Students, teachers, of a government school in Delhi	Structured open-ended self-administered questionnaire, convenience sampling	Summative content analysis with frequency counts via SPSS	Not specified	Awareness of tobacco dangers vs legislation knowledge, Barriers: lack of awareness, indifferent attitudes, High willingness for campaign participation after training	0
57	Vikrant Mohanty^67^	E-cigarettes use behaviour, perceptions and barriers among Indian adults: pilot qualitative research study	2018	Tobacco Induced Diseases	Qualitative (pilot)	42 adult e-cigarette users in Delhi, India	Semi-structured, in-depth interviews (audio-recorded)	Thematic analysis of verbatim transcripts	Not specified	Flavour and taste; perceived as safer alternative; nicotine moderation; cost and availability debates; lack of awareness on legal status; use in places where smoking banned	1
58	Shuba Kumar, Rani Mohanraj, ThailavathyVaidhyalingam, Subhiksha Chakkaravarthi, Badri Thiruvenkatachari^68^	Parental Experiences on Learning About and Caring for Children with Cleft Lip and Palate: A Qualitative Study from South India	2024	Cleft Palate Craniofacial Journal	Qualitative (Grounded Theory)	11 mothers & fathers of children with CLP attending a specialty hospital in Chennai	In-depth, semi-structured interviews; audio-recorded; verbatim transcription	Grounded theory coding and category development	Grounded theory framework (inductive)	Learning about CLP	0
59	Sharath Asokan, Geetha Priya Pollachi-Ramakrishnan, Shruthi Nambi, Sudhandra Viswanath^69^	Piagetian's principles on moral development and its influence on the oral hygiene practices of Indian children: An embedded mixed-method approach	2022	International Journal of Paediatric Dentistry	Embedded mixed-method	50 Indian children aged 7–11 years	Phase 1: Telephonic interviews on Piagetian moral scenarios; Phase 2: Semi-structured interviews on oral hygiene	Content analysis for Phase 1; thematic analysis for Phase 2	Piaget's moral development theory	Comparison of heteronomous vs. autonomous morality Autonomous morality linked to better oral hygiene habits	0
60	Arthi Veerasamy, Jeffrey D. Gage, Ray Kirk^70^	Head teachers' views of oral health education in schools in Tamil Nadu, India	2018	Health Education Journal	Qualitative descriptive	10 head teachers from public and private schools in Tamil Nadu	Semi-structured interviews (audio-recorded, transcribed verbatim)	Thematic coding/analysis	Not specified	Lack of oral health education in schools, Barriers: low recognition, academic overload, competing health priorities, policy inequities. Teachers recognize the importance of oral health education	14
61	Viswanath S, Asokan S, Pollachi-Ramakrishnan G^72^	First dental visit of children – A mixed-method approach	2021	International Journal of Paediatric Dentistry	Sequential mixed-methods	4543 parents of children under 6 in Namakkal district, Tamil Nadu, India	Phase 1: Cross-sectional questionnaire survey, Phase 2: Semi-structured face-to-face interviews with 10 parents	Phase 1: Descriptive statistics Phase 2: Thematic analysis	Not specified	FDV experiences, Barriers to child dental care, Solutions for early dental visits	0
62	Priyanga Chandrasekaran, Priyadharshini Ragavane, Bhargavi K, VikneshanMurugaboopathy, Senthil Murugappan^71^	Dentamatics, a Board Game for Oral Health Education Using the Health Belief Model: A Qualitative Study	2025	Health Education &Behavior	Qualitative	12 schoolchildren (age not specified) in Puducherry, India	Post-game semi-structured interviews; audio-recorded	Thematic content analysis	Health Belief Model	Increased oral health awareness, Interactive learning fosters engagement, Enjoyment and willingness to repeat,Clarifications about dental visits and braces	0
64.	Sequeira M, Naughton F, Velleman R, Murthy P, D'souza J, Pacheco MG, Kamat AK, Gadiyar A, Sanjeevan V, Jain L, Nadkarni A.^73^	Perspectives of smokers, smokeless tobacco users and cessation practitioners in India: A qualitative study	2024	Archives of Psychiatric Nursing	Qualitative (in-depth semi-structured interviews)	Conducted in Goa, India; n = 23 tobacco users (smoked and smokeless) and n = 13 healthcare/cessation practitioners.	Semi-structured in-depth interviews	Thematic analysis	socio-ecological framework	SLT perceived as more culturally acceptable than smoked tobacco; initiation/continuation shaped by multi-level factors; emotionally framed messages about harms to loved ones seen as potentially more motivating for quitting; practitioners described resource gaps and need for tailored cessation supports.	0
63.	Mohanty S, Behera D, Tripathy S, Jena M, Behera MR, Panda B^74^	Prevalence, risk factors, and parental perspectives of dental caries in children in Odisha: A mixed-method study	2024	Clinical Epidemiology and Global Health	Mix-Method	419 school children aged 6–14 years from rural areas of Cuttack and Jagatsinghpur, districts, Odisha	qualitative interviews with 14 parents and 12 teachers.	quantitative data: Descriptive statistics, binary/multiple logistic regression, thematic analysis for qualitative interviews.	Not specified	Overall dental caries prevalence ≈60 %, requent sugar/chocolate consumption, and lower socioeconomic status; parents showed limited awareness about prevention	0

The qualitative studies explored diverse themes. Many focused on the cultural normalization of tobacco use, cessation challenges, and myths about oral health among specific groups such as youth, coronary patients, and vulnerable populations.^16^^,^^25^^,^^26^^,^^28^, ^29^, ^30^^,^^57^^,^^61^ Others addressed early childhood caries, caregiver experiences, parental anxiety during dental procedures, and oral hygiene practices among children^23^, ^24^, ^25^^,^^33^^,^^49^^,^^58^^,^^59^^,^^74^ Barriers faced by vulnerable populations, including individuals with disabilities, caregivers of cancer patients, and tribal groups, emerged as significant concerns^21^^,^^44^^,^^46^ Additionally, studies captured perspectives of dental professionals and educators, including pediatricians and faculty, who reported limited training, time constraints, and systemic challenges in oral health promotion^60^ A smaller set of studies examined psychosocial consequences of oral conditions such as edentulism and potentially malignant disorders, as well as broader policy discourse on tobacco control and fluorosis.^30^^,^^31^^,^^39^^,^^64^

The mixed-methods studies often used surveys alongside interviews to explore the implementation and perception of tobacco cessation interventions in hospitals, rural health programs, and national schemes^15^^,^^36^^,^^37^^,^^39^^,^^42^^,^^48^^,^^53^^,^^55^ Tools such as e-learning modules and text-based cessation messages were assessed for feasibility, showing promise but also revealing barriers related to digital literacy and contextual relevance. Several studies assessed oral health service utilization, highlighting low awareness, financial constraints, and logistical barriers in tertiary and rural settings.^19^^,^^40^^,^^45^^,^^50^^,^^53^ In pediatric research, mixed-methods approaches were used to examine links between feeding practices, ECC, and caregiver nutrition knowledge.^14^ Academic stress, job satisfaction, and curricular innovation in dental education were also key focus areas.^35^^,^^36^ These studies triangulated findings through descriptive or inferential statistics along with thematic analysis, offering nuanced insight into patient behavior, system readiness, and policy implementation. The number of citations per study ranged from 0 to 64 based on Scopus, PubMed, Web of Science, and hand-search sources. While many recent articles had lower citation counts, studies addressing broader public health themes or published earlier demonstrated higher academic visibility, suggesting growing relevance and reach of such research (Table 1).

**Fig. 2:**
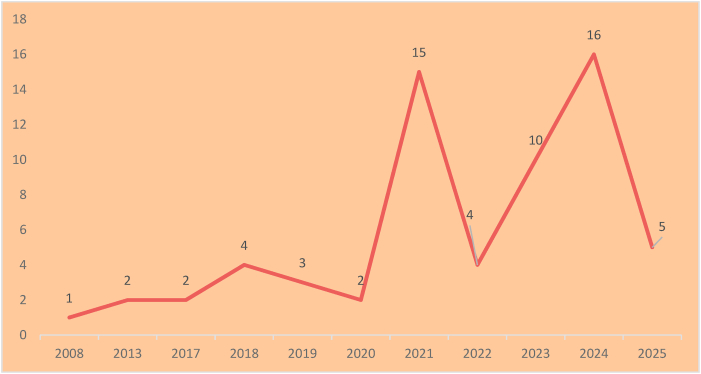
Distribution of eligible studies according to year of publication.

**Fig. 3:**
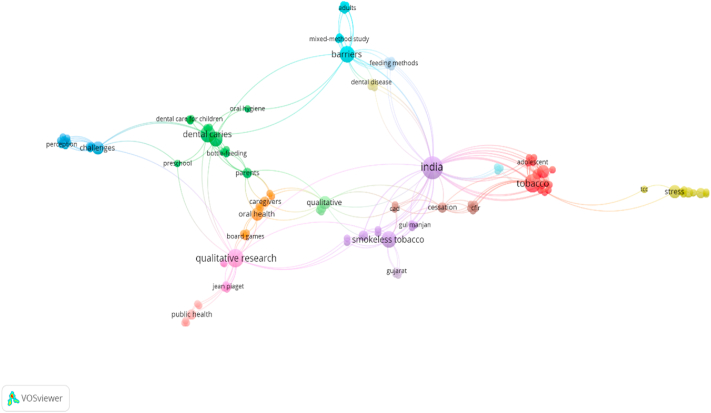
Keywords Co-occurrence Network of Qualitative Research in oral health in India.

The trajectory of publications over the years was plotted on a graph. The data shows the majority were published between 2021 and 2025 (n = 50, 78 %).^10^^,^^12^, ^13^, ^14^^,^^17^^,^^19^^,^^21^^,^^23^^,^^24^^,^^26^, ^27^, ^28^^,^^30^, ^31^, ^32^, ^33^, ^34^, ^35^, ^36^^,^^38^, ^39^, ^40^, ^41^, ^42^^,^^44^^,^^45^^,^^47^, ^48^, ^49^, ^50^^,^^52^^,^^54^^,^^55^^,^^57^, ^58^, ^59^, ^60^^,^^62^, ^63^, ^64^, ^65^, ^66^^,^^68^^,^^69^^,^^71^, ^72^, ^73^, ^74^(Fig. 2). The keyword co-occurrence network of qualitative research in oral health in India is visualized using the VOS Viewer network map (Fig. 3) illustrates the conceptual structure of the field by highlighting frequently co-occurring terms extracted from the titles and abstracts of included studies. From the processed list of 156 keywords that met this criterion, the largest connected set had 118 items, which formed the final visualization network. Prominent keywords such as “tobacco cessation,” “oral hygiene,” “children,” and “behavior” appeared most frequently and were central nodes in the network. These clusters reflect dominant thematic areas, with dense connections suggesting the purple cluster highlights regional and cultural dimensions of smokeless tobacco use in India, particularly in Gujarat, focusing on cessation practices. The green cluster pertains to early childhood oral health, including issues like dental caries, bottle feeding, and oral hygiene among preschoolers. The red cluster centers on tobacco use among adolescents, reflecting behavioral health concerns. The blue cluster explores broad perceptions and challenges, likely relating to patient or provider experiences. The orange cluster emphasizes caregiver involvement and the use of interactive tools like board games to improve oral health awareness. The pink cluster signifies contributions of qualitative research in public health, referencing theoretical frameworks such as Jean Piaget, suggesting developmental or behavioral insights. The light blue cluster focuses on barriers faced by adults, using mixed-method approaches to examine access issues, feeding methods, and dental disease. The brown cluster is grounded in implementation science, referencing tools like CFIR and possibly CAD for planning or diagnostics. Finally, the yellow cluster deals with stress and tobacco cessation counseling (TCC), indicating a psychological and behavioral health focus. The visualization offers insight into how research topics are conceptually linked, helping identify both saturated themes and potential gaps in the qualitative dental research landscape in India (Fig. 3).

## 4 Discussion

This is a pioneer review conducted by combining a literature search and bibliometric analysis focused exclusively on qualitative research conducted within the field of oral health in India. By mapping existing literature and visualizing thematic and conceptual linkages, this review provides a foundational understanding of how qualitative inquiry is being utilized to explore oral health challenges in the Indian context. Unlike previous narrative or topic-specific reviews, this study offers a panoramic view of national-level publication trends, research priorities, and underexplored areas across a diverse range of populations and oral health.

Globally, qualitative methods are recognized as indispensable for understanding policy implementation, health care delivery, dental education, health literacy, caregiver perspectives, clinician decision-making, and cross-sectoral collaborations. Some low- and middle-income countries, notably South Africa, Kenya, and Brazil, have successfully fostered qualitative health care research by strengthening training, securing funding, developing collaborative networks, and employing rigorous methods.^75^, ^76^, ^77^ While this review shows a growing interest in qualitative dental health research in India, especially in the last five years, its depth and volume remain limited, reflecting a nascent discipline in comparison to the global landscape. Despite India having the highest number of dental schools in the world (n = 329) and producing approximately 7175 postgraduates and 27618 undergraduates annually, ^78^ a vast pool of dental professionals who could contribute to strengthening qualitative dental health research remains under-engaged. Qualitative research in field of dentistry in India is still in its incipient stage, with fewer than one article published per month. Approximately one-third of these studies employ a mixed-methods approach, and nearly half do not use qualitative methods at all to investigate dental health issues in India, especially when we exclude those related to tobacco. This underscores a significant capacity gap in conducting meaningful qualitative dental health research in our country. Methodologically, these studies predominantly rely on basic tools, such as interviews and focus group discussions, while lacking analytical rigor, theoretical depth, and reflexivity, reflecting a broader institutional failure to prioritize and foster qualitative inquiry. This gap is especially significant in understanding the determinants of dental caries and periodontal disease, two of the most prevalent non-communicable diseases in both India and globally.^79^

Critically comparing themes across studies, it is evident that tobacco use and cessation dominate the qualitative agenda.^10^, ^11^, ^12^^,^^26^, ^27^, ^28^, ^29^^,^^37^^,^^52^, ^53^, ^54^, ^55^ While this is understandable given India's public health burden, it has resulted in thematic saturation, with less attention to other pressing issues like geriatric oral health,^38^ psychosocial effects of chronic oral conditions,^25^^,^^31^^,^^46^ and barriers in oral healthcare access for marginalized groups.^21^^,^^40^^,^^50^ For instance, studies on caregivers of children with disabilities or cancer patients revealed distinct emotional and logistical challenges^21^^,^^41^^,^^49^ yet such vulnerable groups remain underrepresented in the broader research corpus. Likewise, while a few studies explored academic stress, curricular gaps, or perceptions of dental educators,^18^^,^^24^^,^^35^^,^^36^ these remained descriptive rather than theory-driven.

The use of theoretical frameworks was notably sparse across the included studies. Only a handful explicitly reported using established models such as the Consolidated Framework for Implementation Research (CFIR),^10^ Transtheoretical Model,^12^^,^^37^^,^^55^ Grounded Theory,^24^^,^^41^ or Andersen's Behavioral Model^42^ Most studies lacked reflexivity or theoretical sampling, limiting their capacity to generalize or extend knowledge. This omission hinders the interpretive depth and transferability of findings. For example, while several studies identified fear, misinformation, or access barriers as deterrents to dental care, few examined these through a conceptual lens that could reveal deeper systemic or cultural patterns.^10^^,^^24^^,^^37^^,^^41^^,^^42^

Addressing the gap in the volume and quality of qualitative dental health research in India requires urgent and sustainable efforts from all stakeholders. The logical first step would be to identify the barriers and facilitating factors that can influence the adoption of this paradigm shift within the Indian dental research landscape and to suggest actionable recommendations on their basis.

Several systemic barriers hinder the development and integration of qualitative research methodology in India. A prevailing bias toward quantitative paradigms, along with misconceptions that qualitative methods lack scientific rigor, continues to restrict the field's growth. These perceptions are further amplified by insufficient exposure to and limited formal training in qualitative methodologies at the postgraduate level, resulting in a shortage of skilled researchers in this area. The lack of experienced mentors worsens the situation. Furthermore, issues of institutional indifference and the absence of incentives to conduct qualitative research persist. Together, these factors contribute to the underdevelopment, undervaluation, and underutilization of qualitative research.

Despite these entrenched barriers, there are emerging signs of a shift within the Indian dental research community towardsvalue-based patient-centered care, which aligns naturally with the core principles of qualitative enquiry. India's vast and diverse dental research workforce if equipped with proper training and institutional support, cansignificantly expand the scope and quality of qualitative studies. The country's rich sociocultural landscape and a large, heterogeneous population offer fertile ground for in-depth exploration of oral health behaviors, access to care and patient experiences. Funding opportunities from the government and private sectors are increasingly becoming accessible for health research, with increasing receptiveness to qualitative and mixed-methods research. Certain peer-reviewed journals are beginning to support the dissemination of qualitative work. International collaborations such as the NIHR,UK-funded CORE Project are helping to bridge the capacity gaps through activities such as workshops, webinars, training, mentorship and methodological development. This initiative involves qualitative and mixed-method research exploring oral health inequalities and oral health system reforms across four LMICs, including India. These global partnerships can catalyze local research development.^80^

### 4.1 Strengths and limitations of the review

This review represents the first attempt that critically analyze the contemporary landscape of qualitative methods in oral health research in India. By utilizing data from bibliometric analysis, this study mapped the published literature to identify publication trends, temporal patterns, and both the dominant and unexplored areas within this field. Our review provides valuable insights that can inform future research directions and support the revision of postgraduate dental curricula to incorporate qualitative research. However, the review is not without limitations. A potential limitation of our review is database selection bias, as only two scientific databases are included in the bibliometric analysis and the lack of sufficient location data for accurate zonal classification and recommending this for future work. Consequently, relevant studies published in other databases and grey literature may have been overlooked. Moreover, we did not undertake a critical appraisal of included studies, which could have highlighted methodological weaknesses of the published work and suggested measures to improve the quality of future qualitative research studies.

## 5 Recommendations

There is a pressing need to broaden the scope of qualitative research in oral health in India by adopting advanced methodologies and engaging diverse population groups to gain deeper insights into the social determinants of oral health. To support this expansion, several priority research areas are recommended where qualitative methods can be effectively applied. Some of these areas include exploring oral health inequities and access to care, especially among underserved communities; understanding cultural beliefs and oral health practices that shape health behaviors; and examining oral health literacy to develop more effective communication strategies. Qualitative approaches can also provide rich insights into patient experiences and satisfaction with dental care, identifying key barriers and enablers to service utilization. Investigating school oral health programs through the perspectives of students, parents, and teachers can help improve their implementation and impact. Research on dentist-patient communication can support more empathetic, culturally responsive care, while studies focusing on policy implementation and stakeholder perspectives can reveal how oral health initiatives function in real-world settings. Also, exploring attitudes toward preventive dentistry can guide the creation of community-aligned, sustainable oral health interventions.^81^

It is essential to integrate formal training in qualitative methodologies into our postgraduate dental curricula to strengthen qualitative acumen among early-career oral health researchers. Dedicated coursework should encompass research design, while training programs should focus on research design, data collection techniques (such as interviews, focus group discussions and participant observation), analytical strategies (including thematic analysis or grounded theory) and critical reflexive practices. The adoption of standardized reporting guidelines such as Consolidated Criteria for Reporting Qualitative Research (COREQ) can enhance transparency, methodological rigor and the credibility of published studies.^82^ Encouraging mixed-methods research can also enrich scientific output by integrating the contextual depth of qualitative inquiry with the generalizability of quantitative approaches.

Capacity–building initiatives must extend beyond training students to include faculty development programs, hands-on workshops, and cross-disciplinary collaboration, particularly with the fields of public health, sociology, and anthropology. Strengthening the peer-review ecosystem is essential for sensitizing journal editors and reviewers to the nuances of qualitative research. Academic institutions and professional organizations should actively support qualitative research through scholarships, dedicated journal sections, research grants, and by recognizing qualitative studies in academic promotion criteria. Furthermore, facilitating international collaborations and supporting participation in global funding opportunities can significantly enhance the research capacity, infrastructure, and visibility of qualitative dental research. These recommended steps can establish a strong foundation for high-quality, patient-centered research that addresses the complex realities of oral health care in India.

## 6 Conclusion

This review presents the first comprehensive overview of qualitative research in Indian dentistry, highlighting encouraging growth but persistent gaps in methodological rigor, theoretical depth, and thematic scope. While most studies center on tobacco use and pediatric oral health, areas such as geriatric care, psychosocial experiences, and access to dental services remain underexplored. Building qualitative capacity through education, collaboration, and mentorship, supported by strong institutional commitment and standardized reporting, is essential to advance high-quality, contextually relevant, and patient-centered research that can strengthen oral health research and practices in India.

## Contribution of authors

AM- Conceptualization, methodology, bibliometric analysis, writing – original draft review & editing.

BD- Writing- original draft, review & editing.

MM- Supervision, Writing-review & editing.

## Ethical considerations

This study is a critical review and/or bibliometric analysis, not involving human participants or animal subjects, hence it doesn't require ethical approval.

## Patient’s/guardian’s consent

Not applicable, as this is a bibliometric analysis of publicly available data and does not involve human participants or animal subjects.

## Funding

This research did not receive any specific grant from funding agencies in the public, commercial, or not-for-profit sectors.

## Declaration of competing interest

The authors declare that they have no known competing financial interests or personal relationships that could have appeared to influence the work reported in this paper.
